# Special Issue “Virus-Like Particle Vaccines”

**DOI:** 10.3390/v12080872

**Published:** 2020-08-10

**Authors:** Monique Vogel, Martin F. Bachmann

**Affiliations:** 1 University Hospital for Rheumatology, Immunology, and Allergology, University of Bern, 3010 Bern, Switzerland; monique.vogel@dbmr.unibe.ch; 2 Department of BioMedical Research, University of Bern, 3010 Bern, Switzerland; 3 Nuffield Department of Medicine, Centre for Cellular and Molecular Physiology (CCMP), The Jenner Institute, University of Oxford, Oxford OX3 7BN, UK

Virus-like particles (VLPs) have become a key tool for vaccine developers and manufacturers. They can be broadly used to develop prophylactic as well as therapeutic vaccines in a vast number of indications for human as well as companion animals and animals for food production. An additional use of VLPs is to tune the type and duration of immune responses. In this Special Issue “Virus-like Particles Vaccines”, essentially all of these aspects and applications are discussed and various aspects of VLP vaccinology are highlighted, including VLP characterization. The manuscript by Irene Gonzales-Dominguez et al. is an interesting example, where six different biophysical methods were assessed and compared for the characterization of HIV-1-based VLPs produced in mammalian and insect cell platforms [1]. An important role for VLPs in recent development has been their use as platforms to display antigens. In this context, Ina Balke and Andris Zeltins made an interesting contribution with respect to plant virus-derived VLPs as display platforms [2] as well as Kara-Lee Aves et al. describing the very popular Tag/Catcher system to display antigens on VLPs [3]. As expected, most of the manuscripts focused on the development of prophylactic vaccines in humans. Many VLPs are still developed against classical pathogens, such as influenza virus, norovirus, hepatitis B or E, human cytomegalovirus and human papilloma virus. Peter Pushko and Irina Tretyakova give an interesting outlook for the development of VLP-based vaccines against H7N9 influenza [4] while Arturo Cérbulo-Vazquez et al. present reports on medical outcomes in women that became pregnant after immunization with a VLP-based vaccine against Influenza H1N1 during the 2009 pandemic [5]. Maria Malm et al. make the interesting observation that simultaneous immunization with a multivalent norovirus VLP-based vaccine induces better immune responses than sequential vaccination, reminding readers of the old concept of original antigenic sin [6]. Joan Kha-Tu Ho et al. describe the classical use of HBsAg as vaccine against hepatitis B as well as a novel display platform used e.g., in the malaria vaccine RTS,S [7]. Yike Li et al. describe a novel and interesting VLP-based vaccine against Hepatitis E, currently registered in China [8]. Human Cytomegalovirus has been a long-standing vaccine target with little success. Michela Perotti and Laurent Perez describe an interesting novel VLP-based vaccine designed by structural approaches to combat this virus [9]. Virally sexually transmitted diseases (STDs) are often resistant to current therapeutic treatments. Human papillomavirus (HPV) is the most common sexually transmitted infection and some HPV types are the main causes of cervical cancers. Rashi Yadav et al. present an interesting review on a single VLP-based L2 vaccine which elicit a strong protective immune response against many different types of HPV types [10]. VLPs may not only be used to immunize human prophylactically but also animals. An interesting example for a new animal vaccine candidate is described by Fangfang Wu et al., who describe a VLP-based vaccine against Sudan Virus which is immunogenic in mice and horses [11]. An essential factor for all prophylactic vaccines is their ability to induce long-lived antibody responses, a problem discussed by Bryce Chackerian and David Peabody [12]. Therapeutic vaccines are a new and important emerging topic, covered by vaccines against cancer, atopic dermatitis and cat allergy. Jerri Caldeira et al. give a general introduction to the use of VLPs for the treatment of cancer [13]. John Foerster and Aleksandra Moleda present the concept of displaying cytokines on the surface of VLPs in order to induce anti-cytokine antibodies for the treatment of chronic disease. They use IL-13 as an example [14]. Franziska Thoms et al. finally present the concept of immunizing cats against Fel d 1, the major cat allergen in humans. This reduces Fel d 1 levels in cats and here they demonstrate that this improves the interaction of the allergic cat owner with his cat, as the two can spend more quality time together due to reduced allergic symptoms [15].
